# Mental and functional disabilities in patients with chronic pain

**DOI:** 10.1192/j.eurpsy.2025.817

**Published:** 2025-08-26

**Authors:** O. O. Khaustova, A. E. Asanova

**Affiliations:** 1 Medical Psychology-Psychosomatic Medicine and Psychotherapy, Bogomolets National Medical University, Kyiv, Ukraine

## Abstract

**Introduction:**

Pain has a significant impact on a person’s quality of life, limiting general activity (work, social interaction, problems with family relationships, hobbies/interests, self-care ability), contributing to the development of mental disorders such as depression and anxiety.

**Objectives:**

Our study aimed to determine the level of anxiety, depression, and functional disabilities caused by chronic pain among outpatients in order to further dynamic research of the long-term consequences.

**Methods:**

The study group included 85 outpatients with chronic pain. As a part of psychiatric screening, the HADS depression and anxiety scale was used to study psychopathological symptoms. The WHO self-questionnaire WHODAS 2.0 was used to study the dysfunction caused by chronic pain.

**Results:**

The study found that a significant part of patients with chronic pain had symptoms of anxiety 38% and depression 46% of varying severity. The medial WHODAS 2.0 score among all patients with pain and patients with comorbid depression and anxiety was 23.62 (95% CI: 21.19-24.63), 32.80 (95% CI: 30.33-35.16), and 34.35 (95% CI: 32.11-37.60), respectively. Disability was significantly higher in patients with depression (1.72, p <0.01) and anxiety symptoms (1.60, p <0.01) than in patients with chronic pain without anxiety and depression.

**Conclusions:**

Effective treatment of chronic pain requires a comprehensive approach using psychotherapeutic, psychopharmacological, and physical therapy.

**Disclosure of Interest:**

None Declared

